# Novel Silver-Plated Nickel-Coated Graphite Powder with Excellent Heat and Humidity Resistance: Facile Preparation and Performance Investigation

**DOI:** 10.3390/molecules27134007

**Published:** 2022-06-22

**Authors:** Xin-Kun Lv, Jin-Gang Yu

**Affiliations:** College of Chemistry and Chemical Engineering, Central South University, Changsha 410083, China; csulvxinkun@126.com

**Keywords:** silver plated nickel-coated graphite, electroless plating, silver plating, shielding effectiveness, conductivity

## Abstract

Nickel-coated graphite (Ni/C) powder has many applications in diverse areas such as paint, print ink, adhesive, conductive rubber, and so on. To increase its stability in harsh environmental conditions, the electroless plating of silver film on Ni/C via ascorbic acid was studied. A silver layer with a thickness of 2.5 μm was successfully plated on Ni/C powder’s surface with an Ag loading of 44.35 wt.%. Silica gel blended with the Ag/Ni/C powder exhibited much higher conductivity under aging conditions of 85 °C and 85% RH for 1000 h than that with pristine Ni/C powder. Further tests showed that the conductivity of Ag/Ni/C powder remained almost unchanged even in an extremely humid and hot condition for 1000 h. Aging tests were carried out for Ag/Ni/C and Ni/C powders under long-term humid and hot conditions (85 °C, 85% RH), in which Ag/Ni/C samples showed much better electromagnetic shielding performance. Due to the excellent properties and reasonable price, the potential applications of Ag/Ni/C in conductive glue and electromagnetic shielding glue could be expected.

## 1. Introduction

Metal powders are one of the most important materials for electronic applications. Nickel powder is one of the most significant electromagnetic shielding and wave-absorbing materials used in electronic applications [1,2]. Nickel-coated graphite (Ni/C) powder, which combines the self-lubrication of graphite and the electromagnetic shielding effectiveness of nickel, has been widely applied in paint, print ink, adhesive, conductive rubber, and so on [3]. Despite their good performance, conventional Ni/C powder cannot meet the requirements of some modern applications in harsh environmental conditions such as hot and humid environments because the nickel layer on the outer surface of conductive products suffers from oxidizing, resulting in a sharp decrease in electrical conductivity. To address this challenge, pure precious materials (e.g., silver, gold, and platinum) with good conductivity may be used instead of nickel [4,5,6]. However, pure precious materials have rarely been applied in practice due to their relatively higher prices [7].

Conductive composite materials with acceptable cost and high performance have therefore been developed [8,9,10,11]. For example, silver-coated copper (Ag/Cu) powder showed better oxidation resistance than copper powder, but the long-term resistance to humidity and heat aging was not good [12]. Due to its relatively higher density, the weight per unit volume of silver-coated nickel (Ag/Ni) was relatively higher, resulting in an increased expense [13]. As a lower-cost alternative to fulfill these deficiencies, adding more proportions of lighter and cheaper materials to the composite seems to be highly desirable [14,15]. In addition, the creation of shielding materials with high performance has become an important prerequisite due to the rapid development of smart, wearable electronic devices [16].

Herein, we proposed a method to directly improve material performance by plating a layer of silver nanoparticles onto conventional Ni/C powder. By combining the good conductivity of silver and the advantages of Ni/C powder, such as low cost and being lightweight, this facile route could provide the possibility of fabricating lightweight, inexpensive, and efficient conductive glue and electromagnetic shielding glue. Furthermore, Ag/Ni/C powder could replace Ni/C powder for more application scenarios. Generally speaking, conductive adhesives include epoxy, acrylic, silicone, and so on. Epoxy adhesives and acrylic adhesives are usually heated to melt, then cooled and solidified. By employing the cross-linking reaction and the agglomeration between two components, A and B, organic silicone adhesives could be solidified. The two-component silicon systems with Ag/Ni/C and Ni/C as conductive and shielding materials were also tested [17,18].

## 2. Experimental Details

### 2.1. Materials

Spherical nickel-coated graphite powder E-Fill™ 2707 (60Ni/40C) was provided by Sulzer Metco Co. Ltd. (Wohlen, Switzerland). Silver nitrate (AgNO_3_), sulfuric acid (H_2_SO_4_), ascorbic acid, ethylenediaminetetraacetic acid disodium (EDTA-2Na), ammonium hydroxide (NH_3_·H_2_O), paraffin and ethyl alcohol were bought from Chuandong Chemical Co., Ltd. (Chongqing, China). Two-component silica gels were provided by Dow Corning Corporation (Midland, MI, USA), namely DOWSIL™ [A: poly(dimethyl-methylvinylsiloxane), B: poly(dimethyl-methylhydrogenosiloxane)]. All chemicals were of analytical grade and used without further purification.

### 2.2. Material Fabrication Procedures

Spherical Ni/C powder was preliminarily treated before further applications. Typically, 40.0 g Ni/C powder was soaked in 5 wt.% of sulfuric acid (H_2_SO_4_) solution for 120 min to remove oxidized layers (It is worth noting that the nickel loss would occur) and then was thoroughly rinsed with deionized water until neutral pH was obtained. The pretreated Ni/C powder was suspended by vigorous stirring in 350 mL of deionized water to prepare a Ni/C powder suspension named M. The main salt solution, named T1, was prepared by mixing EDTA-2Na, silver nitrate (AgNO_3_), and ammonium hydroxide at a molar ratio of 1:6:10. The final concentration of EDTA-2Na in T1 was set to be 0.25 mol L^−1^. An aqueous ascorbic acid solution with a concentration of 1.5 mol/L, named T2, was used as a reducing solution.

For silver plating, 160 mL of the main salt solution T1 and 160 mL of the reducing solution T2 were dropwise and separately added into the suspension M under magnetic stirring at a speed of 200 rpm. The whole process lasted for 4 h; the suspension of Ni/C powder after plating was filtered, rinsed, and neutralized with deionized water. The solid on the filter paper was further washed with alcohol, then collected and dried in an oven for 24 h to obtain 57.6 g of silver-plated Ni/C (Ag/Ni/C) powder.

### 2.3. Characterization Methods

X-ray diffraction (XRD) patterns were recorded on a Rigaku MiniFlex 600 X-ray diffractometer equipped with a copper X-ray tube (Cu-Kα radiation, λ = 0.154 nm) and NaI scintillation detector (Tokyo, Japan). Field emission scanning electron microscopic (FE-SEM) images were acquired on a JEOL JSM-6510LV microscope operated at 20 KV. The actual Ag content of Ag/Ni/C powder was analyzed by an inductively coupled plasma-atomic emission spectroscopy (ICP-AES; Optima 8000, PerkinElmer, Waltham, MA, USA). Particle size distribution analysis was performed with a Mastersizer 2000 laser analyzer (Worcestershire, UK). The functional groups of Ni/C and Ag/Ni/C powders were recorded by Fourier transform infrared spectroscopy (FT-IR; Nicolet 6700, Thermo Fisher, Sunnyvale, CA, USA) in the wavenumber range 400–4000 cm^−1^. A double 85 constant temperature/humidity experiment was carried out on a KSON KTHB-510TBS Programmable Temperature & Humidity Chamber (Taiwan, China). Shielding effectiveness was tested with Flange coaxial combined with vector network analyzer system AGILENT E5071C. Thermogravimetric analyses were performed on a TA Instrument (Model: Hi-Res TGA 2950 thermogravimetric analyzer; New Castle, DE, USA) at a heating rate of 10 °C/min to 800 °C in a flowing air atmosphere.

## 3. Results and Discussions

### 3.1. Loading Amount of Ag

Ag/Ni/C powder (0.1947 g) was dissolved in nitric acid and filtered to remove graphite; then, the filtrate was transferred to a 1000 mL volumetric flask, diluted with deionized water to volume, and measured by an ICP-AES. The actual contents of Ag and Ni in Ag/Ni/C powder were tested to be 44.35 wt.% and 26.92 wt.%, respectively.

### 3.2. Characterization

#### 3.2.1. Investigation of Crystal Structure, Morphology, Composition, and Particle Sizes

**Figure 1:**
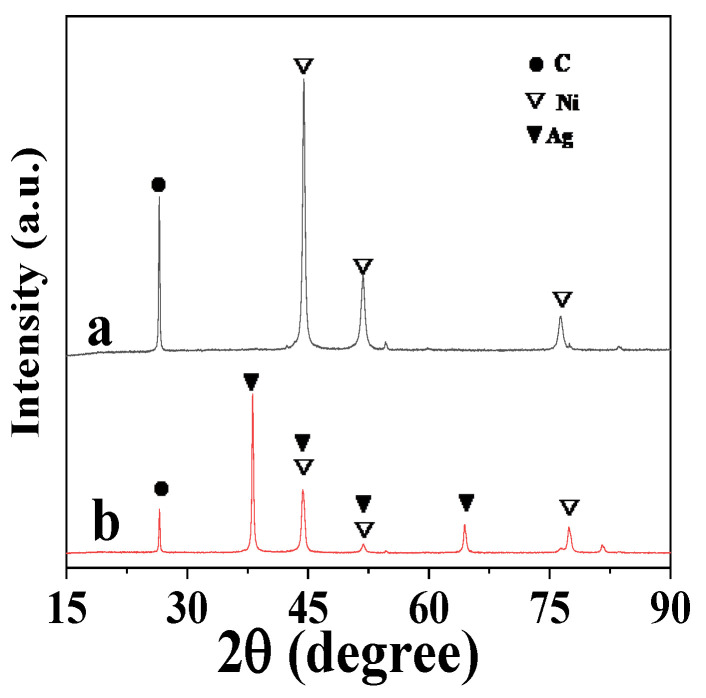
XRD patterns of the samples: (a) Ni/C powders; (b) Ag/Ni/C powders.

XRD was used to verify if the surface silver plating succeeded and the XRD patterns recorded are shown in Figure 1. For the diffraction patterns of Ni/C (Figure 1a), the strongest peak at 2θ of 26.60° corresponds to the characteristic diffraction of graphite (JCPDS cards 75–1621), while the other three peaks at 2θ of 44.49°, 51.84°, 76.37° (JCPDS cards 04-0805) corresponding to pure nickel are also obvious. After silver plating, the peak intensities corresponding to graphite and nickel both decreased significantly, and the three new diffraction peaks appeared at 2θ of 38.12°, 44.31°, 64.46° (JCPDS cards 04-0850) corresponding to pure silver could be observed (Figure 1b). The XRD measurements verified the successful plating of the Ag layer on Ni/C powder.

**Figure 2:**
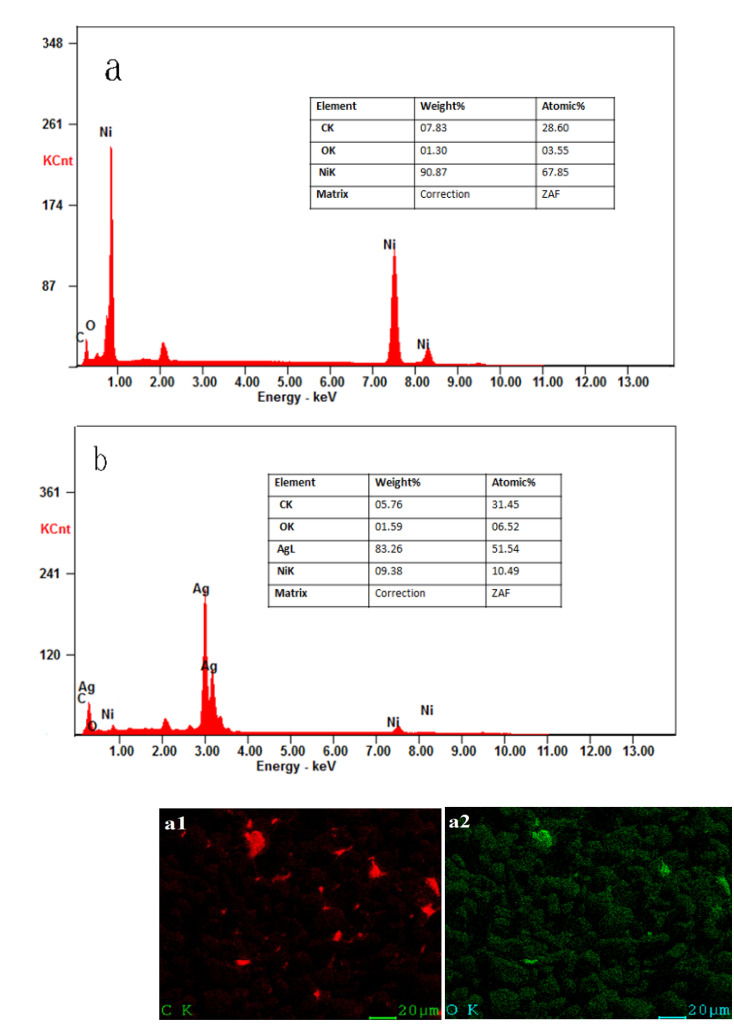

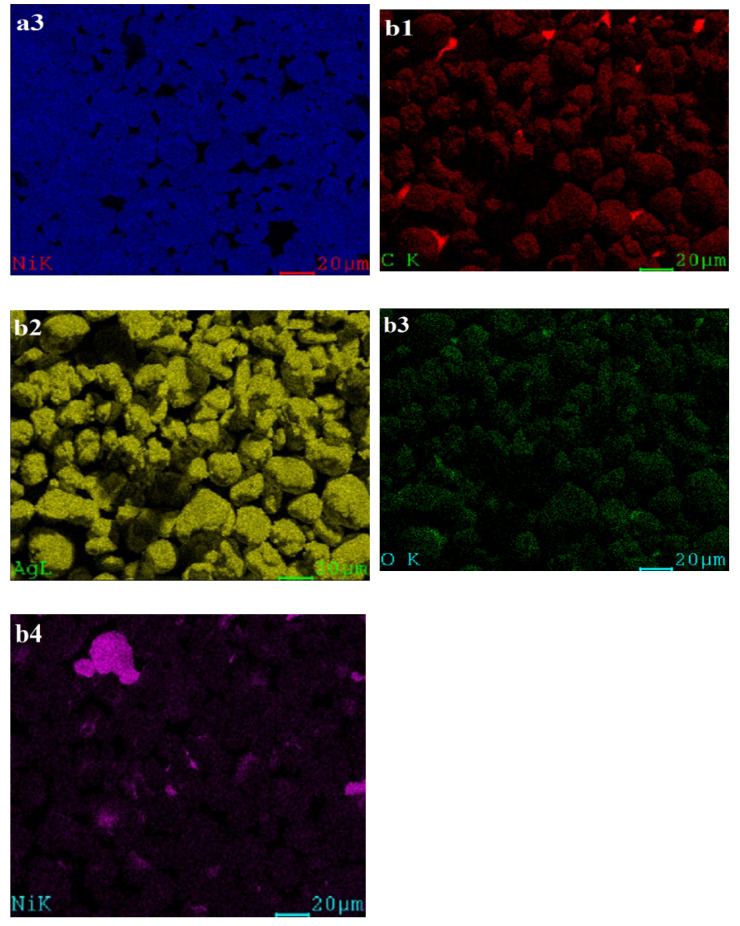
(**a**) EDS of Ni/C powder; (**b**) EDS of Ag/Ni/C powder; (**a1**–**a3**) Elemental distributions of C, O, and Ni in Ni/C powder; (**b1**–**b4**) Elemental distributions of C, Ag, O, and Ni in Ag/Ni/C powder.

The elemental distributions and percentages of the samples were examined by elemental mapping and energy-dispersive X-ray spectrometry (EDS) (Figure 2). EDS analyses indicated that the elemental percentages of C, O, and Ni in Ag/Ni/C powder significantly decreased in comparison with those in Ni/C powder, and the dominant element in the surface layer of Ag/Ni/C was found to be Ag (83.26 wt.%), confirming the successful deposition of Ag on Ni/C powder (Figure 2a,b). Furthermore, elemental mapping analyses demonstrated the presence and uniform distribution of Ag on the powder surface after the coating process, confirming the successful coating of Ag nanoparticles on Ni/C powder (Figure 2(a1–a3),(b1–b4)).

**Figure 3:**
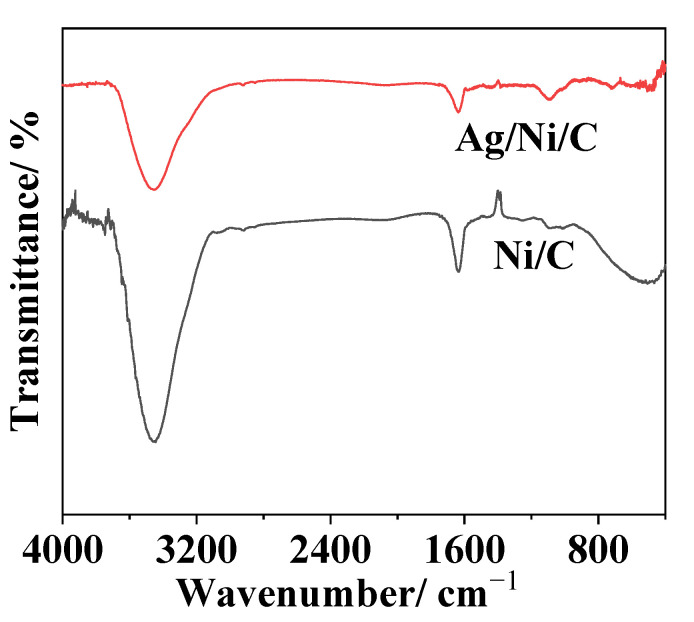
FT-IR spectra of Ni/C and Ag/Ni/C powders.

For FT-IR characterizations, characteristic peaks at 1638 cm^−1^ and 3454 cm^−1^ for the graphite in Ni/C powder could be observed. Especially, the two peaks remained almost unchanged after electroless silver-plating, indicating no further reaction occurred between Ag and Ni/C powder (Figure 3).

#### 3.2.2. Particle Size Distribution Analysis

**Figure 4:**
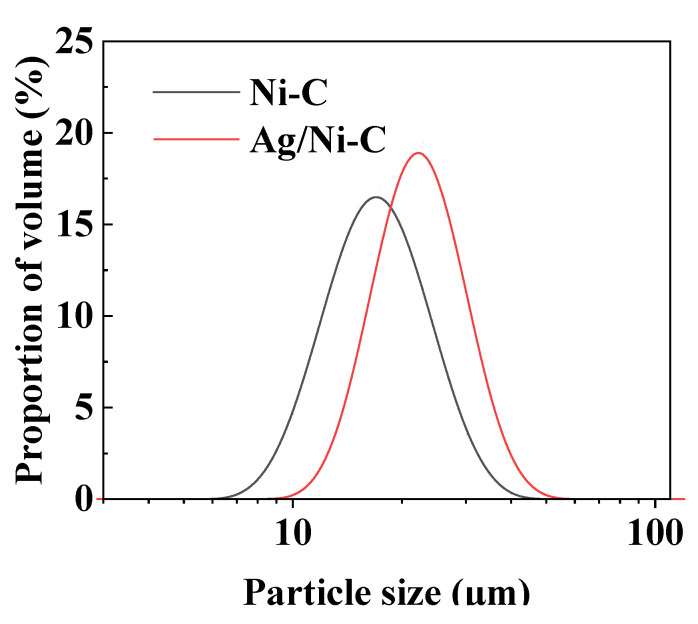
The particle size distributions of Ni/C powder and Ag/Ni/C powders.

A Mastersizer 2000 laser analyzer was used to analyze the particle size distribution of Ni/C and Ag/Ni/C powders. It can be seen from Figure 4 that the particle sizes of Ag/Ni/C powder slightly increased in comparison with those of pristine Ni/C powder.

Based on the data of the particle size distribution, the particle sizes (μm) at below 10% level (d_10_), 50% level (d_50_), and 90% level (d_90_) for both Ni/C and Ag/Ni/C samples were calculated using software Mastersizer 2000. From the data in Table 1**,** it is seen that in comparison with Ni/C powder, d_10_, d_50_, and D_90_ for Ag/Ni/C powder increased by 2.51 μm, 2.56 μm, and 2.24 μm, respectively, indicating the coating thickness was relatively even and stable.

To quantify the cumulative distribution width of particles, the span value and relative span value were proposed. The significance of the span value describes relative dispersion and could be applied to all particle distributions. The values could be calculated by Equations (1) and (2):

Span = (d_90_−d_10_) (1)

Relative span = (d_90_−d_10_)/d_50_ (2)

The calculated span values for Ni/C and Ag/Ni/C powders were 15.08 and 14.81 µm, respectively, while the relative span values were 0.829 and 0.714. The relatively lower values of span and relative span for Ag/Ni/C powder confirmed the presence of a lower percentage of coarser particles. Obviously, the particle size distribution played a significant role in improving the stability of the Ag-coated powder, Ag/Ni/C.

### 3.3. Effects of Reaction Time on Silver Coating

**Figure 5:**
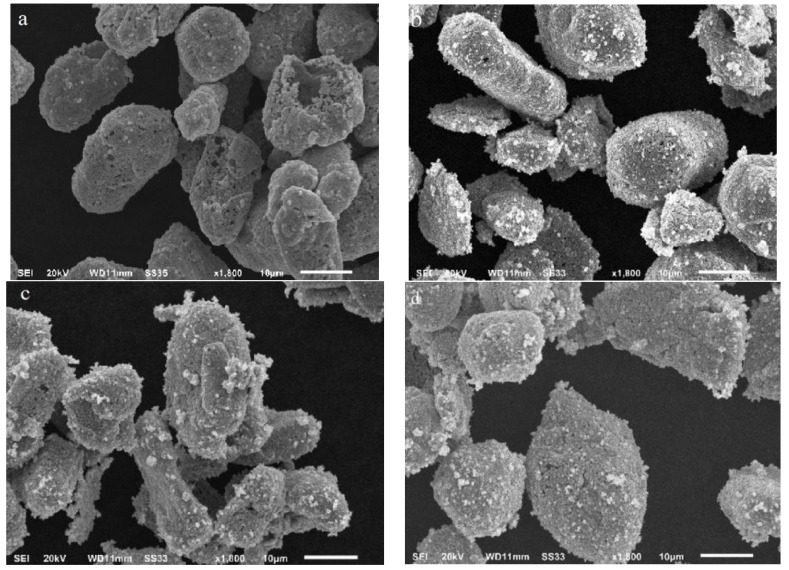

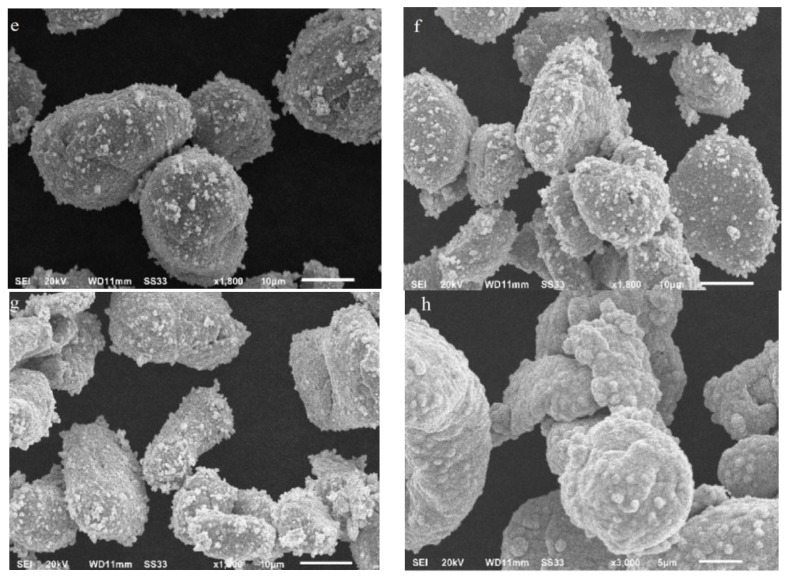
SEM images of the samples during the silver deposition process: (**a**) Surface morphology of E-Fill ™ 2707 Ni/C powder; (**b**–**h**) Surface morphology of the powders at different reaction time intervals: (**b**): 30min; (**c**): 60 min; (**d**): 90 min; (**e**): 120 min; (**f**): 150 min; (**g**):180 min; (**h**): 240 min.

During the silver plating toward Ni/C powder, 5.0 mL of the reaction solution was sucked out every 30 min from 0 to 180 min, then rinsed and dried. The surface morphological changes of the samples were recorded (Figure 5b–g).

Obviously, the nickel layer on the surface of E-Fill ™ 2707 Ni/C powder is not compact, and some exposed carbon cores can occasionally be seen (Figure 5a). From the sample micrographs at different reaction times, it can be seen that with increasing reaction time, the silver dots on the surface of Ni/C powders gradually grew, and dense silver layers would be formed after reaction for 2.5 h. Further increase in the reaction time would lead to deposition of all silver ions in the solution onto the surface of E-Fill ™ 2707 Ni/C powder and result in a too thick silver layer.

### 3.4. Anti-Oxidation Properties of Ag/Ni/C Powder

#### 3.4.1. TG Analyses

**Figure 6:**
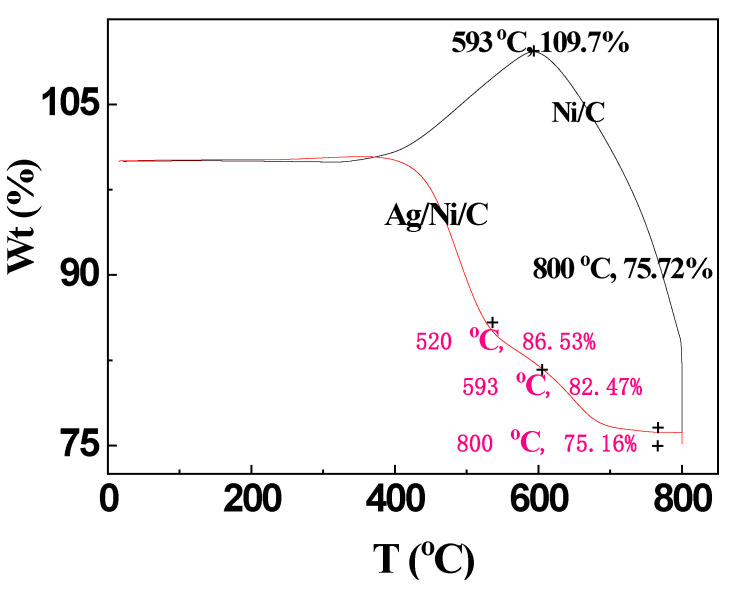
TGA curves of the samples: Ni/C and Ag/Ni/C powders (Balance Gas: Nitrogen 40.0 mL/min; Sample Gas: Air 60.0 mL/min; 1: Ramp 10.0 °C/min to 800.0 °C; 2: Isothermal curing for 120.0 min).

From the TGA data of the two powders under air atmosphere, it can be seen that no obvious weight loss could be observed for Ni/C and Ag/Ni/C powders in the temperature range from room temperature to 400 °C (Figure 6). Under a higher temperature range, nickel would be gradually oxidized to produce nickel oxide, and graphite would be oxidized to produce carbon dioxide. Obviously, the weight increment effect of Ni/C powder was caused by the weight increment due to the formation of nickel oxide being greater than the weight loss of the production of carbon dioxide. Obviously, the percentage of weight increment of Ni/C powder increased to 9.7% in the temperature range of 400–593 °C, and the total weight loss of around 24.28% in the temperature range of 400–800 °C was caused by the pyrolysis of graphite. 

In comparison, weight loss of Ag/Ni/C powder occurred at a higher temperature of 420 °C, indicating silver plating was beneficial to the higher thermo-stability of the powder. Two weight-loss stages can be clearly observed: The weight loss due to the oxidization of graphite in the temperature range of 420–520 °C, which is obviously greater than the weight increment because of the oxidization of nickel; In the higher temperature range of 520–800 °C, the oxidation of nickel powder might occur, while the curve corresponding to the second weight-loss stage is relatively flatter, indicating the oxidation was greatly hindered due to the silver coating on Ni/C powder. At higher temperatures (>593 °C), the combustion of carbon in Ni/C powder occurred, and the gradual weight loss of the sample could be observed. The weight percentage of Ni/C powder was around 75.7% after 800 °C, whereas the Ni content was 60%, indicating Ni was gradually oxidized at high temperatures [18].

According to TGA analyses, Ag/Ni/C powder exhibited a significant positive effect on passivating the oxidization of the nickel layer; therefore, the excellent conductive and shielding properties of Ag/Ni/C powder would be guaranteed.

#### 3.4.2. Effects of Heating Temperature on Ag/Ni/C Powders

As mentioned before, TGA curves showed that when the temperature exceeded 400 °C, the powders would be oxidized in the atmosphere. To investigate the effects of temperature on the morphological change of Ag/Ni/C powders, the heat treatment of Ag/Ni/C powder in a muffle furnace was performed. Some powders were taken out at different temperatures (400 °C, 500 °C, 600 °C, and 700 °C), transferred into a vacuum desiccator, and cooled naturally to room temperature.

**Figure 7:**
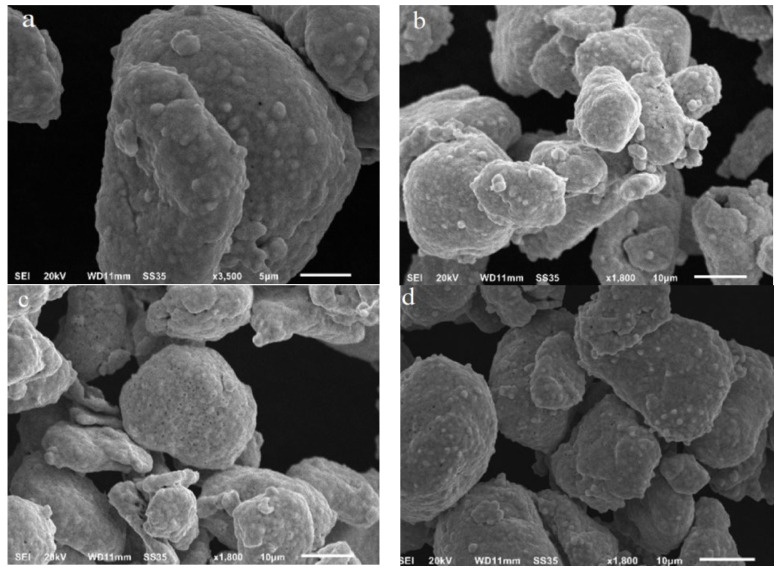
SEM images of Ag/Ni/C powders at diffident temperature: (**a**) 400 °C; (**b**) 500 °C; (**c**) 600 °C; (**d**) 700 °C.

The surface changes of these samples treated at high temperatures were investigated by FE-SEM. The appearance of some individual molten holes in the outer silver layer at 400 °C could be observed, and the inner Ni/C layers would be exposed to air (Figure 7a). As the temperature increased to 500 °C, the silver layer began to collapse. We clearly observed that the “molten holes” increased a lot (Figure 7b), and the inner layers were mostly exposed to the atmosphere, resulting in more serious oxidation [19]. As the heating temperature increased to 600 °C and 700 °C, more “molten holes” appeared on the surface of the Ag/Ni/C powders, directly leading to the rapid oxidation of carbon and nickel layers in the core (Figure 7c,d). It is further pointed out that the oxidation mechanism of Ag/Ni/C powder is that the micron level silver coating on the Ni/C surface was firstly melted at 400 °C due to the “size effect,” and then surface shrinkage occurred, and the inside “cores” were exposed. With an increase in heating temperature, the shrinkage of the silver layer was more prominent, and more and more “molten holes” would be formed to expose mostly cores which were finally oxidized in air.

### 3.5. Hydrothermal Resistance and Electromagnetic Shielding Effectiveness of Ag/Ni/C and Ni/C Powders

#### 3.5.1. Evaluation of the Electrical Conductivity

**Figure 8:**
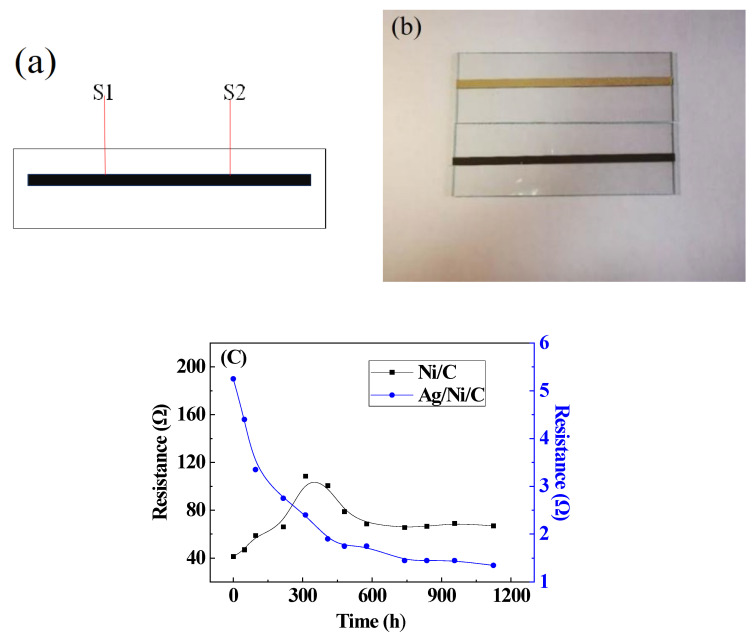
Long-term conductivity tests of Ag/Ni/C and Ni/C powder-based membranes: (**a**) A sketch of the tested membrane; (**b**) Tested samples; (**c**) Conductivity tests.

To evaluate the long-term conductivity of Ag/Ni/C powders and Ni/C powders as conductive adhesive products, two-component silica gels were used as the experimental samples. The two components include A and B components: A component is a poly(dimethyl-methylvinylsiloxane) and B component is poly(dimethyl-methylhydrogenosiloxane). The A and B components were blended at a mass ratio of 10:1. Ag/Ni/C powders and Ni/C powders were used as conductive materials (60 wt.%) to prepare two kinds of conductive adhesive, respectively. After the conductive adhesive was mixed and defoamed, it was then applied onto a glass slide by a dispenser to form a strip-shaped membrane (width: 2.5 mm; thickness: 100 μm). The prepared samples were placed in room temperature conditions for 48 h. The crosslinked membrane was treated in an environment of constant temperature and humidity (85 °C and 85% RH). The sample was taken out at different time intervals, and the resistance value of conductive silica gel was analyzed using a HIOKI 3451 resistance tester. The distance between the points (S1 and S2) is 10 mm (Figure 8a).

The conductivity of conductive silica gels blended with Ni/C powder or Ag/Ni/C powders were tested by long-term effects of exposure to high temperature and humidity; the results of tracking resistance tests are shown in Figure 8c. Obviously, the samples containing Ni/C powder possessed high conductivity. In comparison, the initial resistance of conductive silica-containing Ag/Ni/C powder was relatively smaller, which was only about one-eighth of the value obtained at conductive silica gel-Ni/C powder, indicating Ag/Ni/C powder possessed higher conductivity. In addition, even after being placed in an environment of 85 °C and 85% RH for 1100 h, no significant changes in the conductivity of the conductive silica gel-Ag/Ni/C powder could be observed during the long-term aging process. Undoubtedly, the dense silver layer on the surface of conductive Ni/C particles provided effective protection against harsh environmental conditions. After 700 h of exposure to heat-moisture treatment (HMT), the resistance value of the conductive silica gel-Ag/Ni/C powder decreased to about 27% of its initial value, while the conductivity almost did not change significantly later in the hot and humid environment.

On the contrary, the resistance of conductive adhesive containing Ni/C powder increased sharply at the beginning of the tests. We proposed that Ni/C powder might be rapidly oxidized to nickel oxide under hot and humid conditions [20]. As the conductivity of the powder decreased, the shrinkage in the conductivity of conductive silica gel would inevitably occur. After exposure to heat-moisture treatment for 400 h, nickel on the surface of the conductive powder was further oxidized, and the subsequent resistance value tended to be stable at high values of ~80 Ω after 600 h due to the total oxidization of the surface nickel layer.

The results showed that the resistance of conductive silica gels containing either kind of conductive particles (Ni/C or Ag/Ni/C) decreased during the long-term aging process in the hot and humid environment. The main reason might be that silica gel was completely solidified and shrunken in the hot and humid environment, and the contact among the compressed conductive particles became much more interconnected; thus, the resistance greatly decreased [21,22].

#### 3.5.2. The Electromagnetic Shielding Performance

The electromagnetic shielding performance of the two conductive silica gels containing conductive particles (Ni/C or Ag/Ni/C) was measured using a system composed of a vector network analyzer AGILENT E5071C and a flange coaxial fixture. The shielding effectiveness of the blend of paraffin wax and metal powder was measured in the frequency range of 1–18 GHz in accordance with ASTM D7449/D7449M-2008e1 covering the whole microwave frequency range [16,23,24,25].

The paraffin and different powders (Ni/C powders pre- and post-HMT for 1000 h, Ag/Ni/C powder pre- and post-HMT for 1000 h) with a mass ratio of 6:4 was added to a beaker, which was then placed in an oven and heated to 80 °C for 20 min. After the paraffin was completely melted, the baker was taken out, and the mixture was stirred evenly at room temperature (RT). Under continuous stirring, the mixture gradually became viscous; then, the beaker was transferred into the oven and heated to 80 °C for another 20 min. The whole operation of continuous stirring at RT and heating in the oven was repeated three times, and a uniformly dispersed mixture could be obtained. The mixture was transferred into a ring-mounted mold and pressed into a ring-shaped architecture. The sample obtained possessed a controlled thickness of 2.0 ± 0.02 mm (The outer diameter of the sample: 7 mm; the inner diameter: 3 mm).

**Figure 9:**
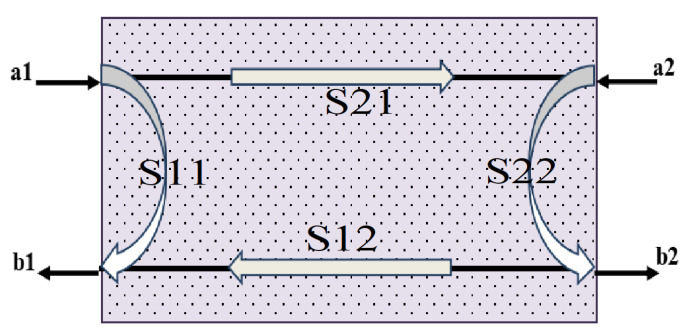
Proposed EMI shielding mechanism.

An Agilent E5071C vector network analyzer (VNA, Agilent E5071C; Agilent Technologies Inc., Santa Clara, CA, USA) was used to analyze the electromagnetic shielding performance of the samples in the frequency range of 1–18 GHz. Four scattering parameters, S11, S12, S21, and S22, could be obtained (Figure 9).

The S11 and S22 parameters have the same value and represent the refection, and a1, a2, b1 and b2 represent incident and feedback signals of terminals 1 and 2, respectively. The S12 and S21 parameters are the same and represent the transmission coefficient.

The total shielding effectiveness (SE, dB) of material can be obtained by the sum of reflection loss (SE_R_, dB), absorption loss (SE_A_, dB), and multiple reflection loss (SE_M_, dB). Compared to SE_R_ and SE_A_ in this material system, SE_M_ is relatively smaller and can be ignored, a related equation could be listed as Equation (3):

SE ≈ SE_A_ + SE_R_ (3)

The relationship between SE_R_, SE_A_ and S11, S21 could be expressed as follows:

SE_R_=−10 lg[(1 − |S_11_|^2^)] (4)

SE_A_=−10 lg[(1 − |S_11_|^2^)/|S_21_|^2^] (5)

**Figure 10:**
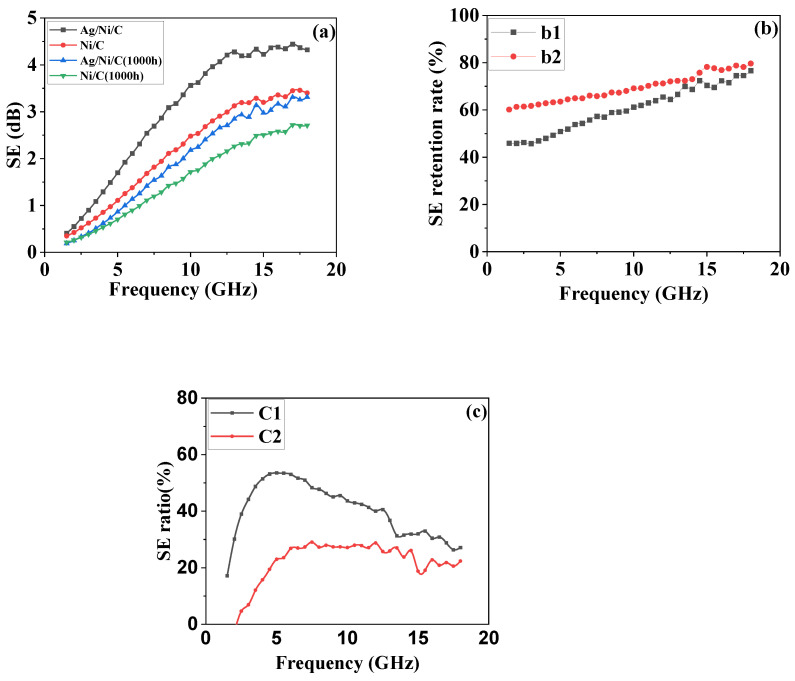
Shielding effectiveness tests of Ag/Ni/C and Ni/C powder-based specimens: (**a**) Shielding effectiveness of four powder materials (Ni/C powders pre- and post-HMT for 1000 h, Ag/Ni/C powders pre- and post-HMT for 1000 h); (**b**) SE retention rate of Ag/Ni/C post- and pre-aging (b1), SE retention rate of Ag/Ni/C post- and pre-aging (b2); (**c**) SE ratios of Ni/C powder to Ag/Ni/C powder pre-aging (c1) and post-aging (c2).

We calculated SE according to the above Equations (3)–(5) [15,26,27,28,29], and the results are listed in Figure 10a. Obviously, as frequency increases, SEs of the two powder materials pre- and post-HMT increase as a result. It’s also worth noting that Ag/Ni/C powders exhibited relatively better shielding performance than Ni/C powders regardless of whether they were exposed to HMT or not.

We also compared the shielding values of the powders before and after exposure to HMT at the same frequency by using the SE retention rate (%; SE_post-aging_/SE_pre-aging_ × 100%) of the sample post- and pre-aging, and the calculated results are shown in Figure 10b. As the frequency increases, the shielding difference of SE retention rates of the two powder materials pre- and post-aging gradually decreases. Additionally, Ag/Ni/C powder exhibits a higher SE retention rate than Ni/C powder at the same frequency. The two powder materials post-aging basically exhibits 80% of the shielding effectiveness of pre-aging powder materials at the frequency of 18 GHz.

The SE ratios of the two powder materials pre- and post-aging at different frequencies were compared and analyzed (Figure 10c). The SE ratios (%; SE_Ni/C_/SE_Ag/Ni/C_ × 100%) of Ni/C powder to Ag/Ni/C powder pre-aging (curve c1) and post-aging were calculated (curve c2). From the calculated data, SE ratios of the two powders, no matter pre- or post-aging, increased with an increase in frequency over the frequency range 1–5 GHz, indicating that the samples exhibited better-shielding performance at lower frequencies. Subsequently, the SE ratios of the two powders decreased with an increase in frequency over the frequency range of 5–18 GHz. Obviously, the SE ratios of Ni/C powder to Ag/Ni/C powder pre-aging were higher than those of post-aging, indicating Ag/Ni/C powder was more resistant to corrosion than Ni/C powder.

**Figure 11:**
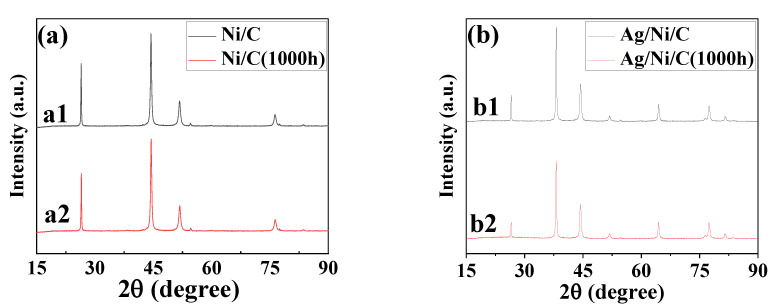
XRD patterns of the samples: (**a**) Ni/C powders pre- and post-HMT for 1000 h; (**b**) Ag/Ni/C powders pre- and post-HMT for 1000 h.

XRD was used to verify the crystal structure change of Ni/C and Ag/Ni/C after HMT for 1000 h. From Figure 11a,b, the XRD results indicated that there was no crystal structure change for both samples, pristine Ni/C and Ag/Ni/C powders, after HMT1000 h.

**Figure 12:**
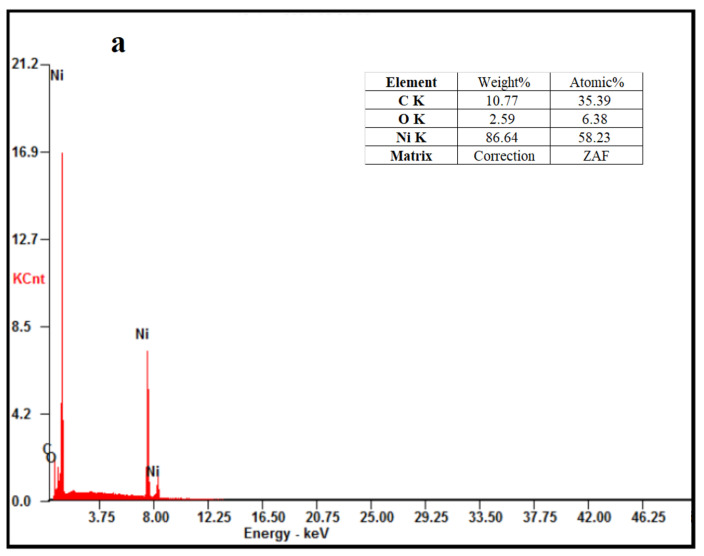

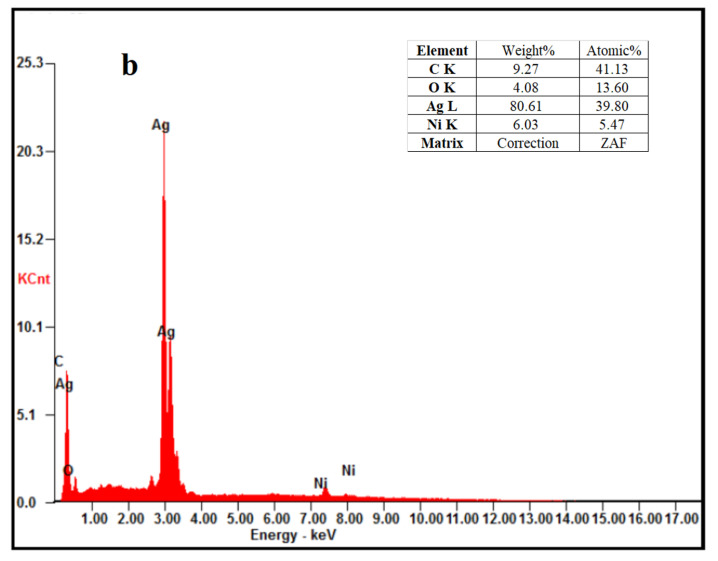
(**a**) EDS of Ni/C powders post-HMT for 1000 h; (**b**) EDS of Ag/Ni/C powders post-HMT for 1000 h.

The EDS analyses results showed that the oxygen (O) contents in Ni/C and Ag/Ni/C powders (Figure 12a,b) greatly increased after HMT 1000 h in comparison with those of as-prepared samples (Figure 1a,b), indicating that both the Ni/C and Ag/Ni/C powders were oxidized. As AgO is conductivity while NiO is non-conductive, Ag/Ni/C powder exhibited better electromagnetic shielding performance than Ni/C, indicating it possessed higher moisture and thermal resistance.

## 4. Conclusions

A novel conductive material, Ag/Ni/C powder, with a silver content of 44.35 wt.% and silver coating layer thickness of 2.5 μm, was prepared by using ascorbic acid as the reducing agent. Under long-term humid and hot aging conditions (85 °C, 85% RH), the silica gel-based conductive adhesive containing Ag/Ni/C powder exhibited relatively more stable conductivity in comparison with the control group containing Ni/C powder. Besides, Ag/Ni/C powder pre- and post-exposure to long-term humid and hot aging conditions (85 °C, 85% RH) also showed better electromagnetic shielding performance than Ni/C powder.

## Figures and Tables

**Table 1:** Particle size distributions of the samples.

Sample	d_10_ (μm)	d_50_ (μm)	d_90_ (μm)
**Ni/C**	12.15	18.19	27.23
**Ag/Ni/C**	14.66	20.75	29.47

## Data Availability

Not applicable.

## Sample Availability

Samples of the compounds are not available from the authors.
